# Engagement of private healthcare providers for case finding of tuberculosis and diabetes mellitus in Pakistan

**DOI:** 10.1186/s12913-020-05217-2

**Published:** 2020-04-19

**Authors:** Shifa Salman Habib, Sana Rafiq, Wafa Zehra Jamal, Shaikh Muhammad Ayub, Rashida Abbas Ferrand, Aamir Khan, Syed Mohammad Asad Zaidi

**Affiliations:** 1 Community Health Solutions, 9th Floor, Al-Tijarah Building, Main Shahrah-e-Faisal, Karachi, Pakistan; 2 London School of Hygiene and Tropical Medicine, Keppel St, Bloomsbury, London, WC1E 7HT UK; 3 Interactive Research & Development, 4th Floor, Woodcraft Building, Plot No. 3 & 3-A, Sector 47, Korangi Creek Road, Karachi, Pakistan

**Keywords:** Tuberculosis, Diabetes, Co-morbidity, Healthcare

## Abstract

**Background:**

The rising co-epidemic of tuberculosis (TB) and diabetes mellitus (DM) is a challenge for constrained health systems in low and middle-income countries. Diabetes is a known risk factor for tuberculosis and associated with poor tuberculosis treatment outcomes, while tuberculosis is associated with worsening glycemic control. We investigated the performance of bi-directional TB and DM case finding approaches through a private-sector engagement model in Karachi, Pakistan.

**Methods:**

Between July 2016 and July 2018, private health care providers were engaged to generate referrals for bi-directional TB and DM screening at private diagnostic and treatment centers in Karachi, Pakistan. Individuals diagnosed with TB underwent glycated hemoglobin (HbA1c) testing at the time of anti-tuberculous treatment initiation and at three -month follow up stage. All individuals with a history of diabetes or random blood sugar of greater than 200 mg/dl were screened for TB using a chest X-ray and Xpert MTB/RIF.

**Results:**

A total of 6312 persons with tuberculosis were tested on HbA1c at treatment initiation, of whom 1516 (24%) were newly diagnosed with DM. About one third of those with HbA1c in the diabetic range (≥ 6.5%) at baseline were found to have a normal HbA1c (< 5.7%) result at 3-month follow-up. A total of 3824 individuals with DM, of whom 2396 (63%) were known cases and 1428 (37%) were newly identified with random blood sugar > 200 mg/dl, underwent chest x-ray and Xpert MTB/RIF testing, with 321 (13.4%) known and 54 (3.8%) new diabetics respectively identified with tuberculosis.

**Conclusion:**

This study demonstrates a high yield of TB and DM through bidirectional screening and the feasibility of engagement of private sector in finding missing cases of tuberculosis and diabetes. Given the high prevalence of undiagnosed DM in individuals with TB tuberculosis patients, there is a need to scale-up DM screening within TB programmes. Increased awareness of the high risk of TB among individuals with DM is needed among private health providers and screening for TB among diabetics should be strongly considered.

## Background

The global burden of Diabetes Mellitus (DM) has doubled from 1980 to 2017, posing a significant economic burden on health systems globally [1]. The International Diabetes Federation has estimated that the number of people living with DM worldwide will rise from 463 million in 2019 to 700 million by 2045 [2]. Tuberculosis (TB) remains the leading cause of death from a single infectious agent with an estimated 1.5 million deaths in 2018 [3].

There is increased recognition of the synergy between DM and TB [4, 5]. Currently, an estimated 1 million people globally are present with TB-DM comorbidity, higher than the number of patients with TB-HIV co-infection [4]. DM increases the risk of developing TB three-fold and is associated with delayed sputum conversion, treatment failure, relapse and death [6]. TB in turn is associated with worsening glycemic control.

Based on available evidence, the International Union against TB and Lung Disease launched a Call to Action in 2014 that emphasized upon the significance of bi-directional screening and joint management of TB and DM [7]. However, implementation of these recommendations has been challenging in countries such as Pakistan where the private sector contributes to a majority of health services delivery. There have been very limited private-sector engagement initiatives that have addressed the dual burden of TB and DM. We piloted a bi-directional screening project that developed a network of trained private-providers, linked to diagnostic and treatment centers for improved patient outcomes [8].

In this study we investigated the outcomes of the scale-up of a TB-DM bi-directional screening program in the private-sector in Karachi, Pakistan, modelled on the World Health Organization’s (WHO) Collaborative Framework for care and control of tuberculosis and diabetes [9]. In addition to expanding the geographic coverage of the program, our current study also addresses the limitation of the pilot by using glycated hemoglobin (HbA1c) instead of random blood sugar (RBS) test as the diagnostic test for DM among individuals with TB.

## Methods

### Study setting and design

A retrospective cross sectional study in which, bidirectional screening for TB and DM was offered to clients attending private TB diagnostic and treatment centers “*Sehatmand Zindagi (SZ)* (healthy life) centers and community screening camps in Karachi, Pakistan between July 2016 and July 2018. Karachi is Pakistan’s most populous city and the country’s economic hub with an estimated 75% of all health services availed in the private sector [10]. Pakistan has the fourth highest burden of DM globally with a current estimated prevalence of 17.1% in the adult population. An estimated 8.5 million adults are living with undiagnosed DM [2]. Pakistan is also ranked fifth among high tuberculosis burden countries with an estimated 36% case notification gap [3].

The SZ centers, located in low middle income neighborhoods of Pakistan operate as a social business, providing free TB diagnostics, chest X-ray and Xpert MTB/RIF, and treatment. Revenue is generated through other laboratory tests and radiology services. All TB cases are notified to the National TB Control Program (NTP). Each center has established linkages with other health providers in the vicinity including those in the informal health sector. The center has a dedicated team who engage private health providers and facilitate referral of individuals with presumptive TB to the centers. Clients are also able to self-refer to centers. Community based screening camps are conducted by trained community health workers employing mobile X- ray vans.

### Recruitment of individuals with TB for DM testing

Individuals presenting at SZ Centers and screening camps with a previous history of TB or with signs and symptoms of TB were referred to the TB arm of screening where they were tested for DM if TB diagnosis was established. TB screening was conducted using digital chest X- rays with CAD4TB 5 (version 4.12.0) software for automated scoring Individuals with presumptive TB were defined as those with a threshold CAD4TB score of 70, who then submitted a sputum sample for Xpert MTB/RIF testing. A positive Xpert MTB/RIF testing result or a strong indication at clinical evaluation of the CXR and symptoms lead to the diagnosis of bacteriologically positive or clinical TB respectively. All individuals identified with TB underwent HbA1c testing at anti-tuberculous treatment (ATT) initiation. Those who gave consent also had a 3-month HbA1c follow up. According to the recommendation of 2009 American Diabetes Association (ADA), an HbA1c < 5.7% is classified as normal, 5.7–6.4% as pre-diabetes, and ≥ 6.5% as diabetes [11]. Individuals who were diagnosed with diabetes at the SZ Center were counselled and referred to their general practitioner for further management.

### Recruitment of individuals with DM for TB testing

Individuals attending the community camps and SZ Centers were verbally screened for history of diabetes. Those identified with DM or those presenting with signs and symptoms of DM were referred to the DM arm of screening where they were subsequently tested for TB if DM was diagnosed. If no known history of DM was found, they were offered a point of care glucose test. Individuals with an RBS > 200 mg/dl or history of diabetes, were tested on chest X-ray and Xpert MTB/RIF.

### Data management and analysis

We analyzed retrospective data of 10,136 Individuals who participated in the TB-DM bidirectional screening project funded by the World Diabetes Foundation. Under this project, data was recorded using a custom-built mobile-phone application at community camps, A customized web-based laboratory management system (LMS) software was used to book tests and enter screening data at the centers. Both the mobile and web-based applications were integrated with the Central Management Information System with auto generated reports to track key project metrics. The data recording and reporting systems included several data validation checks to ensure data-accuracy. Field supervisors and project management staff were responsible for overall data-validation and accuracy including reporting to the NTP. Baseline characteristics of participants were described using means and medians and the prevalence of DM and TB were calculated. The association of outcomes (DM and TB) with a prior defined factor was explored using logistic regression. A comparative frequency analysis was conducted for HbA1c values at baseline and after 3 months of ATT for a subset of TB patients. All data was analyzed using Stata version 13.0 (StataCorp, Texas, USA).

### Ethical approval and consent

An ethical approval was deemed unnecessary for this study by The Institutional Review Board (IRB) at Interactive Research and Development (IRD) under the IRB exemption category 7 under 45 CFR 46.101(b). The IRB is registered with the U.S. Department of Health and Human Services (DHHS), Office for Human Research Protections (IRB#00005148). A Verbal consent was obtained from participants before conducting blood glucose, HbA1c and Xpert MTB/RIF tests. This study was part of a larger study (IRB approval number IRD_IRB_2016_08_001). De-identified data from the project was used for data analysis.

## Results

### Diabetes screening in individuals with TB using glycosylated hemoglobin (Hba1c) testing

**Fig. 1:**
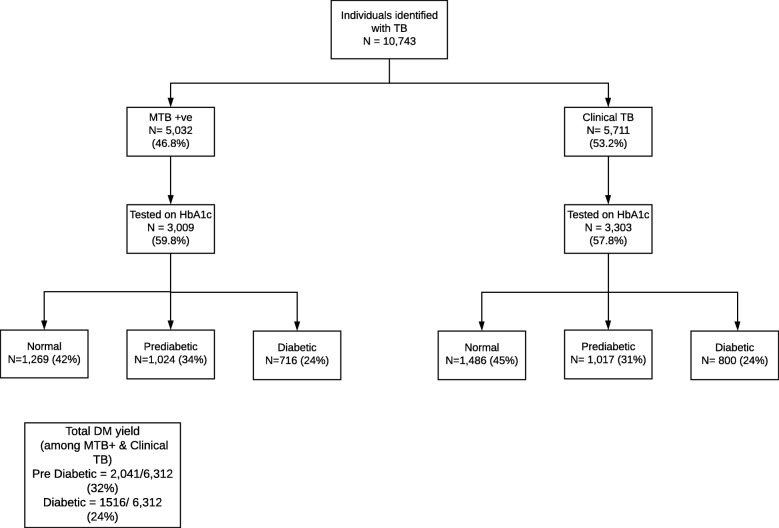
Uptake of diabetes screening among individuals with tuberculosis

A total of 5032 individuals had a positive *Mycobacterium tuberculosis* (MTB) result on Xpert MTB/RIF testing, while 5711 were identified with clinical TB (Fig. 1). Of these, 6312 were tested for DM using HbA1c. The uptake of HbA1c testing was 59.8 and 57.8% in persons with bacteriologically positive and clinical TB respectively (Fig. 1). A majority (5093) were new TB cases. Among those screened for DM, 2041 (32%) had pre-diabetes and 1516 (24%) had newly diagnosed DM (Table 1). Of those newly diagnosed with DM 25% were male and 35% were aged 40–59 years. The number needed to test (NNT) to make one DM diagnosis among individuals with TB was 4.16 (Table 1).

**Table 1:** Baseline characteristics of individuals with TB tested for diabetes using HbA1c and numbers needed to test (NNT) to identify one case of diabetes

	Total TB Cases Tested for DM n (%)	Normal n (%)	Pre-Diabetes n (%)	Newly Diagnosed DM cases n (%)	NNT^‡^	***P*** Value
**Total**	**6312**	**2755 (44%)**	**2041 (32%)**	**1516 (24%)**	**4.16**	
**Sex**						
Male	3347	1461 **(43.7%)**	1052 **(31.4%)**	834 **(24.9%)**	**4.01**	**0.122**
Female	2965	1294 **(43.6%)**	989 **(33.4%)**	682 **(23%)**	**4.35**	
**Age (years)**						
15–20	885	462 **(52.2%)**	318 **(35.9%)**	105 **(11.9%)**	**8.43**	**< 0.001**
20–39	2407	1309 **(54.4%)**	739 **(30.7%)**	359 **(14.9%)**	**6.70**	
40–59	1750	593 **(33.9%)**	544 **(31.1%)**	613 **(35%)**	**2.85**	
> 60	1270	391 **(30.8%)**	440 **(34.6%)**	439 **(34.6%)**	**2.92**	
**TB diagnosis**						
MTB+	3009	1269 **(42.2%)**	1024 **(34%)**	716 **(23.8%)**	**4.20**	**0.017**
Clinically diagnosed	3303	1486 **(45%)**	1017 **(30.8%)**	800 **(24.2%)**	**4.13**	
**Past History of TB**						
No (New case)	5093	2226 **(43.7%)**	1649 **(32.4%)**	1218 **(23.9%)**	**4.18**	**0.927**
Yes (Re-treatment	1219	529 **(43.4%)**	392 **(32.2%)**	298 **(24.4%)**	**4.09**	

### Screening for TB among individuals with diabetes using Xpert MTB/RIF testing

**Fig. 2:**
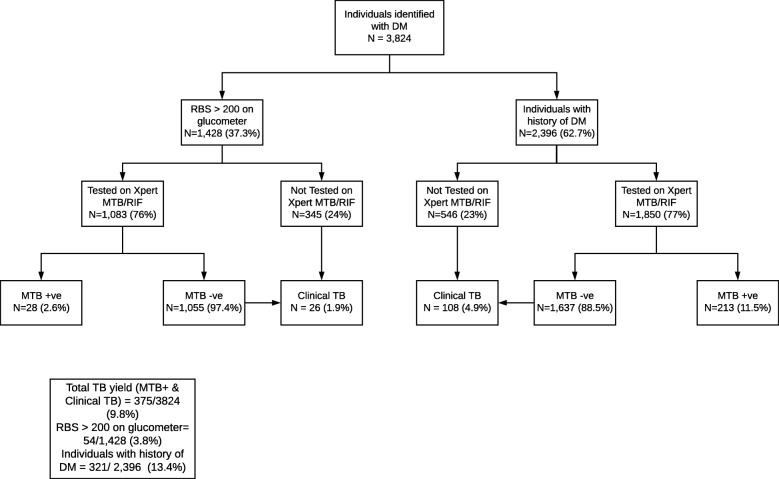
Uptake of tuberculosis screening among individuals with diabetes

A total of 3824 individuals with DM underwent screening for TB at the SZ centers (Fig. 2). Of these, 1428 (37%) had an RBS greater than 200 mg/dl on point of care testing and 2396 (63%) had a known diagnosis of DM (Fig. 2). A majority were males and were aged 40–59 years (Table 2). Among those referred from community camps and partner providers, 66.6 and 52.2% respectively were known DM screened for TB whereas, 33.4 and 47.8% respectively were individuals with RBS > 200 mg/dl. The uptake of Xpert MTB/RIF testing among individuals with RBS > 200 mg/dl and known DM was 76 and 77% respectively (Fig. 2). Of the total individuals with TB detection, 85.6% were known cases of DM and 14.4% were individuals with an RBS > 200 mg/dl who underwent Xpert testing (Table 2).

**Table 2:** Baseline characteristics of individuals with previously diagnosed diabetes and those identified with RBS > 200 mg/dl through screening in the private-sector in Karachi, Pakistan, from July 2016 to July 2018

	Total n (%)	RBS > 200 n (%)	Known case of DM n (%)	***p***-value
**Sex**	3824	1428 **(37.3%)**	2396 **(62.7%)**	< 0.005
Male	2202	777 **(35.3%)**	1425 **(64.7%)**	
Female	1622	651 **(40.1%)**	971 **(59.9%)**	
**Age**				< 0.001
15–20	85	46 **(54.1%)**	39 **(45.9%)**	
20–39	673	328 **(48.7%)**	345 **(51.3%)**	
40–59	1892	696 **(36.8%)**	1196 **(63.2%)**	
> 60	1175	358 **(30.5%)**	817 **(69.5%)**	
**Source of referral**				< 0.001
Community Camps	1288	430 **(33.4%)**	858 **(66.6%)**	
Others (hospitals, NGOs)	331	108 **(32.6%)**	223 **(67.4%)**	
Sefl-referred	903	268 **(29.7%)**	635 **(70.3%)**	
Private provider	1302	622 **(47.8%)**	680 **(52.2%)**	
**TB diagnosis**				< 0.001
No TB detected	3449	1373 **(39.8%)**	2076 **(60.2%)**	
MTB + ve	241	28 **(11.6%)**	213 **(88.4%)**	
Clinical diagnosis	134	26 **(19.4%)**	108 **(80.6%)**	
**Total TB**	375	54 **(14.4%)**	321 **(85.6%)**	

Known diabetes (OR 4.63, CI 3.21–6.66) was the strongest associated factor in final adjusted models for MTB detection (Table 3).

**Table 3:** Predictors for TB detection among individuals with diabetes tested using Xpert MTB/RIF, visiting TB diagnostic and treatment centers in Karachi, Pakistan (July 2016–July 2018). Significance testing has been done using chi-squared test

	Univariate analysis			Multivariate analysis		
	OR	95% CI	*P* value	OR	95% CI	*P* value
**Gender**						
Male	Ref	Ref	Ref	Ref	Ref	
Female	1.16	(0.91–1.49)	0.237	1.21	(0.94–1.55)	0.133
**Age**						
Age (years)	1.08	(0.92–1.26)	0.343	0.99	(0.83–1.16)	0.890
**Previous History of DM**						
No previous history (RBS > 200)	Ref	Ref	Ref	Ref	Ref	
Known case of DM	4.58	(3.19–6.59)	0.00	4.63	(3.21–6.66)	< 0.0001

### Follow-up HbA1c testing for individuals with TB

Among individuals with TB tested on HbA1C at the ATT initiation, 1077 individuals also underwent follow-up HbA1c testing after 3 months and 514 (47.7%) had an unchanged HbA1c range between the baseline and follow-up tests.

**Fig. 3:**
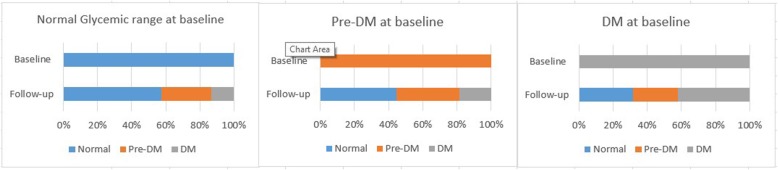
Comparison of HbA1C at baseline and after 3 months of anti-tuberculous treatment at private healthcare clinics in Karachi, Pakistan

Among the 244 individuals with an HbA1c ≥ 6.5% at baseline 64(26%) and 77(32%) had anHbA1c that dropped to 5.7–6.4 and < 5.6% respectively at 3 months. Of the 331 persons with HbA1c in the pre-diabetes range at baseline, 148 (44%) dropped to < 5.6% at 3 months. Of the 502 patients with HbA1c < 5.6% at baseline, 213 (42%) had increased HbA1c ≥ 5.7% at follow-up (Fig. 3).

## Discussion

This study describes findings from the first bi-directional screening programs for TB and DM carried out at scale in the private sector. This project was innovative in its use of CAD4TB for TB screening and the use of HbA1c for initial screening of DM among TB patients.

As in other studies [12–15], we report a high prevalence of TB among persons with DM. We also found that the odds of developing TB were nearly 4.5 times higher in persons with previously diagnosed DM compared to those who were newly diagnosed. It is possible that chronic hyperglycemia may be associated with an increased risk for developing TB. A recent study showed increased TB risk with duration of DM, with a median time to diagnosis for TB of 7 years among those with DM [16].

Individuals with TB were screened for DM using Hba1c. The prevalence identified in our study of pre-DM (32%) and DM (24%) was much higher among individuals with TB than in the general population of Pakistan (17%) [2]. This is consistent with findings from other studies that have reported a high prevalence of up to 35% of DM in TB patients [17]. The susceptibility of older individuals to TB and DM in our study is also consistent with previous studies highlighting the risk and the need for regular screening among older individuals [18–20]. HbA1c testing was repeated for subset of TB patients after 3 months of ATT. Transient hyperglycemia is a common finding among people with TB, with HbA1c levels reverting to the euglycemic range after 3 months of TB treatment [5, 21]. The cause of transient hyperglycemic status is likely multifactorial and due to a combination of inflammation induced by TB, the hyperglycemic effect of ATT, and individual risk of DM [21]. Transient hyperglycemia in individuals with TB makes it challenging to determine the appropriate time for screening to avoid misdiagnosis of DM. Our data suggests that screening at 3-month follow-up may be more appropriate than at ATT initiation, although further drop in Hba1c by end of ATT completion cannot be excluded. However, this needs to be balanced against the risk of potential lost to follow-up. The Union also recommends a follow-up HbA1c at treatment completion to avoid the risk of overdiagnosis of DM [6].

Our study supports the need to scale-up DM testing in existing TB programmes. An advocacy meeting facilitated by our group with stakeholders from NTP, the Diabetes Association Pakistan, the Ministry of Health and other policy makers endorsed recommendations to conduct routine screening for DM among TB patients at TB Basic Management Units (BMUs). These measures are being included as part of the National Strategic Plan for TB control in Pakistan for implementation at the district-level. Pakistan has a well-organized vertical TB program with over 1360 BMUs that provide standardized data surveillance and reporting [22]. Indicators for DM screening can feasibly be added within the existing reporting framework. Similar resources are also planned for allocation for provision of diagnostic services for public-private mix (PPM) implementing partners of the NTP, targeting the private sector.

With an estimated 19.4 million adults with DM in Pakistan, there is an important opportunity for finding undiagnosed cases of TB by incorporating TB screening within DM services [2]. Lack of awareness of the increased risk of TB among individuals with DM, especially in the private sector needs to be therefore urgently addressed. This may be challenging, as unlike TB, there is no vertical disease program targeting non-communicable disease (NCDs) in the country. Pakistan was one of the first low-income countries to formulate a comprehensive National Action Plan to address NCDs, but it is yet to be implemented [23]. Training, incentives for referrals and continuous engagement of private-practitioners may facilitate TB screening in DM services. Our study supports the use of chest X-ray for TB screening in individuals with DM in high TB burden settings. While providers frequently refer patients for screening for diabetic neuropathy, diabetic nephropathy and dyslipidemia, TB could be included as an important complication of diabetes. The use of new technologies such as computer-aided detection software can facilities this process. Multi-disciplinary advocacy efforts including collaboration with diabetes professional associations and the pharmaceutical industry are required to increase awareness of TB-DM co-morbidity, combined with initiatives by NTPs to target individuals with diabetes as a high-risk group to be screened.

### Limitations

It is likely that providers referred diabetics for TB screening that had symptoms of TB or had other risk factors, resulting in selection bias.

The study results may also have been confounded by other risk factors associated with TB among individuals with DM such as access to healthcare, level of glycemic control, compliance with DM medication as well as environmental factors such as smoking. In addition, we did not quantify the duration of DM among those screened for TB.

## Conclusion

This study provides evidence for engagement of private sector in finding missing cases of TB and DM through systematic bi-directional screening approaches. Given the high prevalence of undiagnosed DM in TB patients, there is a need to scale-up the availability of DM testing and treatment services in TB facilities. A high yield for TB was identified among diabetics and pre-diabetics in our study population. There is an urgent need to address the lack of sensitization among the private providers about the risk of TB among diabetics, due to which TB cases among diabetics are frequently missed. Multi-disciplinary advocacy efforts including collaboration with diabetes professional associations and the pharmaceutical industry are required to increase awareness of the TB-DM co-morbidity.

## Data Availability

The datasets used and analysed during the current study are available from the corresponding author on reasonable request**.**

## Abbreviations

TB: Tuberculosis
Xpert: Xpert MTB/RIF test
CXR: Chest x-ray
CAD4TB: Computer Aided Detection for Tuberculosis
DM: Diabetes Mellitus
MTB: *Mycobacterium tuberculosis*
RBS: Random blood sugar
ATT: Anti Tuberculous Treatment
NTP: National TB Control Program
NCD: Non-communicable disease
